# German Review

**Published:** 1891-04

**Authors:** Leo. Breisacher


### Selections from Foreign Journals.

### GERMAN.

By Leo. Breisacher, V. M. D.

### Degeneration of Peripheral Nerves in Animals.

Dr. H. Hamburger.—He examined the nervi mediani and tibialis portici of horses, cattle and dogs, and found in some cases where death was caused by infectious diseases a degeneration similar to the degeneration found in the beri-beri of the human subject. *Monatschriftf. Thierheilkunde No. 11.*

### The Disinfection of Cattle Cars.

P. Canalis. —As the result of experiments of C. the following mode of disinfecting cattle cars has been adopted in Italy: After removal of straw, etc., from the car the walls are scraped with a large spatula especially devised for the purpose and then washed with a ten per cent. acid sololution of sublimates—using a brush with stiff bristles—and finally irrigated with a sublimate sololution of the same strength. The doors are left open until the walls have dried thoroughly. For every car about forty litres of solution are necessary—60 grammes sublimate, and 200 grammes muriatic acid. *Ibid.*

### Mouth Speculum for the Examination and Operation in Dogs and other Small Animals.

Prof. Leschner.—We know of no instrument whose absence in veterinary medicine was more felt than a mouth speculum for dogs and other small animals. Prof. L. has devised a speculum which seems to fill the want in every respect. It is manufactured by Ernst Borman, Vienna Rochusgasse 3. *Ibid.*

### Osteomalacia.

Vet. Winkler, of Grafenan.—Reports that in his district osteomalacia was almost entirely absent in 1889 while in other years this district, as is generally known in Germany, suffers a greater loss from this disease than from all other infectious diseases combined.

He attributes dry weather and an insufficient amount of phosphates and calcium as the principle causes. In former years when the fields and pastures were treated with the offal gotten from the potassium factories in the neighborhood of Grafenan, osteomalacia was unknown in that district. Winkler is of the opinion that in swine the chief cause of osteomalacia is the feeding of potatoes and sour milk. He disputes the idea of some "theorists" that lactic acid plays no role in the production of osteomalacia, and offers the results of several experiments on some fed with milk and sour milk in defense of his view. (It seems to us not at all impossible that the negative results obtained in relation to experimental production of osteomalacia are due mostly to outward circumstances which with care can be overcome.) *Wochenschrift f. Thierheilkunde in Viehzeucht No. 45.*

---

PHOSPHORESCENT PORK.

Vet. Gotteswinter.—Reports an interesting case of phosphorescence of fresh pork, the phosphorescence of the vertebræ was so intense that they resembled pieces of iron at white heat. Microscopically nothing abnormal could be noticed—a microscopic examination was not made. The meat was fed to a dog without any bad effects. About eighty pounds of the meat of the same swine were consumed by different individuals without the production of any effects whatever. In regard to the remarks which G. makes on the production of phosphorescence, etc., the editor of the *Wochenschrift* expresses himself as follows: Hersten who observed phosphorescence of pork thought a micro organism was the cause. Wehenkel and Lahn failed to find an organism. Blanc examined phosphorescent horse meat and determined that the *light* appeared on the partially dried surface before decomposition sets in. In regard to the intensity, B. remarks that the phosphoresence of a piece of meat four inches square is sufficiently strong to make the hands of a watch discernible in a dark room. The duration of the phosphoresence is for horse meat 4—9 days, for beef 1–6, for rabbit meat 1–3 and for dog meat 1–2 days. According to B. phosphoresence is caused by an oval diplococcus (an organism to which the name Micrococcus Pflügeri has been given, has been described and has long been held to be the chief factor in the production of phosphoresence. Reviewer,) which develops in bouillon, urine and gelatine of either neutral alkaline or acid reaction. It does not produce phosphoresence of the nutritive medium. Re-inoculated meat does not show phosphoresence however upon raw meat the organism produces its characteristic action. B. does not think that in the organism itself the phosphoresence is produced but upon the surface of the meat.

Neusch-Basel is of the opinion that the process in the production of *light* in bacteria, wood, plants and insects is identical in all cases with the *light* production of phosphoresence which consists of a slow combination of an organic body with oxygen of the atmosphere. This organic body is not identical with phosphorus. Through the "vital process" of animals and plants certain carbonaceous substances are produced which in combining with oxygen give rise to the *light* phenomena. *Wochenschrift f. Thierheilkunde No. 46.*

---

### Chemical Tuberculin.

Prof. Liebreich, of the University of Berlin, whose discovery of chloral hydrate first brought him into prominence and has given him a world-wide reputation, has discovered a chemical substance which is said to possess all the virtues of Koch's tuberculin. Unlike tuberculin it does not however produce fever nor endanger the life of the patient. Several recoveries of cases of tuberculosis of the larynx, as the result of the application of this substance, have been reported. L. will give the composition mode of preparation, etc., of this new substance at the next meeting of the Berliner Med. Geselloshaft.

